# Effect of Different Types of Adhesive Agents on Orthodontic Bracket Shear Bond Strength: A Cyclic Loading Study

**DOI:** 10.3390/ma16020724

**Published:** 2023-01-11

**Authors:** Irfan Eser, Orhan Cicek, Nurhat Ozkalayci, Mehmet Yetmez, Hande Erener

**Affiliations:** 1 Department of Orthodontics, Faculty of Dentistry, Zonguldak Bulent Ecevit University, Zonguldak 67100, Turkey; 2 Department of Healthcare Management, Boyabat Faculty of Economics and Administrative Sciences, Sinop University, Sinop 57000, Turkey; 3 Department of Mechanical Engineering, Faculty of Engineering, Zonguldak Bulent Ecevit University, Zonguldak 67100, Turkey; 4 Department of Orthodontics, Faculty of Dentistry, Tekirdag Namık Kemal University, Tekirdag 59030, Turkey

**Keywords:** orthodontics, brackets, adhesive agent, bonding, cyclic loading, shear stroke, strength

## Abstract

Bracket failure is one of the most important problems encountered during fixed orthodontic treatment. For this reason, different types of adhesive agents have been developed over the years. Consequently, the aim of this study was to evaluate the shear bond strength of brackets bonded to teeth etched with a conventional acid etching method in a laboratory environment by using different types of adhesive agents and comparing the number of shear strokes. Sixty human maxillary premolars were divided into three groups and Gemini stainless steel metal brackets (3M Unitek, Monrovia, CA, USA) were bonded to all teeth. In *Group 1*, Transbond™ XT Primer (3M Unitek, Monrovia, CA, USA) and Transbond™ XT Light Cure Adhesive Paste composite (3M Unitek, Monrovia, CA, USA) were used. In *Group 2*, BracePaste^®^ MTP Primer (American Orthodontics, Sheboygan, CA, USA) and BracePaste^®^ Adhesive composite (American Orthodontics, Sheboygan, WI, USA) were used. In *Group 3*, Ortho Solo™ Primer (Ormco, Orange, CA, USA) and Grengloo™ Adhesive composite (Ormco, Brea, CA, USA) were used. The samples were subjected to a shear test with a closed-loop controlled, low-cycle fatigue machine with a capacity of 10 N and a crosshead speed of 300 mm/min. The number of shear strokes of the brackets was recorded. According to the Kruskal–Wallis and Mann–Whitney U tests performed on the data obtained, statistically significant differences were found between the groups in terms of the numbers of shear strokes (*p* < 0.05). Significantly higher numbers of shear strokes and higher shear bond strengths were observed in *Group 3* compared with *Group 1* and *Group 2* (*p* < 0.05). There was no statistically significant difference between the numbers of shear strokes for *Group 1* and *Group 2* samples (*p* > 0.05). To conclude the study, it was observed that the type of adhesive used had an effect on the bond strength of the bracket and that the Grengloo™ adhesive agent showed higher shear bond strength. It was observed that BracePaste^®^ Adhesive and Transbond™ XT Light Cure Adhesive Paste adhesive agents had similar shear bond strengths.

## 1. Introduction

Orthodontic brackets are among the most important passive elements of fixed orthodontic mechanics used to transmit the force produced by the force elements to the teeth [1]. Since the beginning of fixed orthodontic treatment, brackets have traditionally been welded to metal bands [2].

Since the introduction of enamel etching by Buonocore [3], the use of composite resin materials for bonding orthodontic brackets has helped to revolutionize orthodontic treatment. A few years later, Newman [4] used this technique for bonding orthodontic brackets, and the direct bonding technique has become routine in orthodontic practice.

There are many factors that affect orthodontic bond strength. In relation to the material, the enamel etching method, bracket design, and type of adhesive agent are reported; related to teeth, the tooth type and fluorosis, are reported; and in relation to environmental factors, blood, saliva, and moisture contamination are reported [5,6,7,8].

The strength of the bond between the bracket and the enamel surface is mainly dependent on the retention mechanism of the bracket base, the adhesive agent, and the tooth surface preparation. Enamel surface treatment, primer solution, and adhesive composite resin are used in the bonding of orthodontic brackets, which are widely used [9].

One of the main goals of fixed orthodontic treatment is to provide an appropriate bond strength between orthodontic brackets and tooth surfaces. The bond should be strong enough to resist the forces that occur during the treatment process and be secure enough to allow removal at the end of the treatment without damaging the tooth enamel [10,11]. Reynolds [12] stated that the tensile bond strength of the brackets should be between 6 and 8 MPa. In addition, there are studies that have determined the limits of shear bond strength to be between 4 and 10 MPa [13,14]. Although the values specified by Reynolds are used as references in many shear bond strength studies, it has been reported that the tensile bond strength values should not be compared with the shear bond strength values [6].

One of the inevitable problems encountered in fixed orthodontic treatments is the failure of the attachment. The failure of the brackets from the tooth surface affects the orthodontic treatment process. The treatment process takes longer and, as a result, the duration of pain or discomfort experienced by patients is prolonged. Bond failure can occur at the adhesive-tooth interface or at the adhesive-bracket interface. Brown [15] reported, in his study, that the failure of bracket attachment should not exceed 6%. However, Almosa et al. [16] reported that the average attachment failure rate in orthodontic treatments is 0.6–28.3%. Khan et al. [7], in their study, revealed that the failure rate of brackets in the posterior region is higher than that of the anterior-region brackets. Menini et al. [17] and Sukhia et al. [18] stated, in their study, that the bracket failure rate was higher in mandibular teeth compared to maxillary teeth.

In vitro bond strength tests provide a guideline for the clinical selection of bracket–adhesive combinations. Bond strength tests are carried out as shear, tensile, and torsion tests according to the force application mode. The use of shear forces has received more attention due to the simplicity of the experimental configuration and simulating bracket failure that occurs during treatment. Tensile or torsion tests, on the other hand, are seen as less relevant for clinical applications. However, the wide distribution of results and the lack of standardization of bond strength test protocols generally preclude reliable results [19,20].

The aim of this study was to compare the shear bond strengths of metal brackets bonded to human maxillary premolar teeth etched by the conventional acid etching method. According to the shear strokes, three different types of adhesive agents were considered for testing under cyclic loading in an in vitro environment.

## 2. Materials and Methods

### 2.1. Ethical Approval and Teeth Used in the Study

Ethics committee approval was obtained for the study, dated 18 November 2020, with decision number 2020/22–18/11/2020, from the Non-Invasive Clinical Research Ethics Committee of Zonguldak Bulent Ecevit University. The sample size calculation was performed in the G*Power 3.1.9.7 program. The effect size was calculated by using the means and standard deviations of the groups. The α error probability was set to 0.05. The power of the study (1-α error prob) was set to 0.95. According to these data, the actual power of the study was calculated to be 95% and the total sample size should have been 42. In the study, 60 human maxillary premolars, 20 in each group, were used. The criteria for tooth selection were the absence of caries, fillings, and restorations, no malformations on the buccal surface, no cracks and fractures on the enamel’s surface, no fluorosis, and no history of tooth bleaching [21].

### 2.2. Storage Conditions of Teeth

The teeth were washed to remove organic residues after extraction. In order to protect the tooth enamel and prevent dehydration and bacterial growth, it was stored in distilled water containing 0.1% thymol in a dark environment and a glass container for a maximum of 6 months. The solutions were renewed monthly [22].

### 2.3. Preparation of Acrylic Blocks and Embedding of Teeth

**Figure 1:**
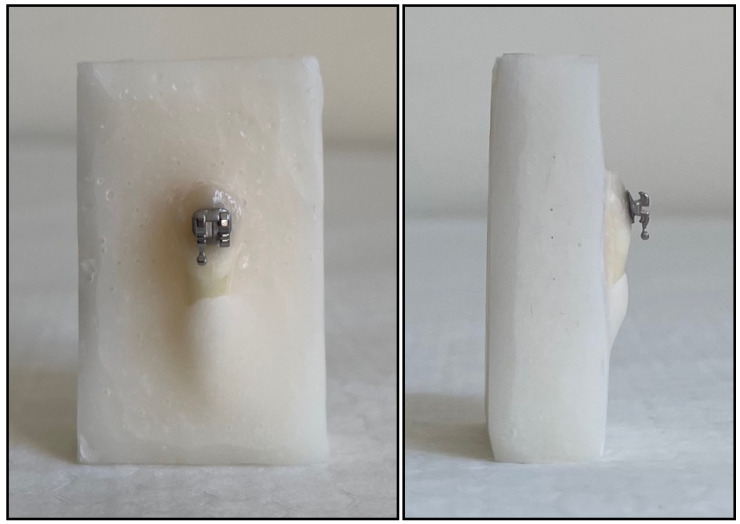
The buccal surfaces of the teeth are embedded perpendicular to the stainless-steel nozzle tip, which will exert the shear force.

Rectangular-prism-shaped molds were used for acrylic block preparation. Vaseline was applied to the molds for the easy removal of the blocks before the teeth were embedded. Autopolymerizing acrylic was prepared and poured into the molds. While the teeth were embedded, care was taken to keep the buccal surfaces exposed from the enamel–cementum junction (see Figure 1). By fixing the samples, a mechanism was created in the test machine in such a way that the nozzle tip stroked perpendicular to the bracket groove [23]. There was no acrylic contact with the buccal surface of the teeth. After the acrylic polymerization, the blocks were taken out of the molds and numbered, and they were then kept in distilled water for 24 h to prevent the tooth enamel from drying out until the test process.

### 2.4. Brackets Used in the Study

In the study, 60 Gemini stainless steel metal brackets (3M Unitek, Monrovia, CA, USA) with 0.022 × 0.028-inch slots were used. The base surface area of the bracket was determined to be 9.61 mm^2^ by learning from the manufacturer.

### 2.5. Adhesive Agents Used in the Study

The adhesive agents used in the study for Group 1, Group 2, and Group 3 are listed below:

*Group 1*:

Transbond™ XT Primer (3M Unitek, Monrovia, CA, USA);

Transbond™ XT Light Cure Adhesive Paste (3M Unitek, Monrovia, CA, USA).

*Group 2*:

BracePaste^®^ MTP Primer (American Orthodontics, Sheboygan, WI, USA);

BracePaste^®^ Adhesive (American Orthodontics, Sheboygan, WI, USA).

*Group 3*:

Ortho Solo™ Primer (Ormco, Orange, CA, USA);

Grengloo™ Adhesive (Ormco, Glendora, CA, USA).

The compositions and weight percentages of the adhesive agents used in Group 1, Group 2, and Group 3 are given in Table 1.

### 2.6. Bonding of Brackets

The buccal surfaces of all teeth were cleaned by applying a pumice–water mixture with a polyture rubber. Afterward, the samples were washed and dried. Then, 37% phosphoric acid gel was applied to the buccal surfaces of the teeth. After waiting for 30 s, it was washed with water for 30 s and dried with air for 15 s. After a chalky white appearance was observed on the surface of the teeth, a thin layer of each group’s own primer was applied to the enamel with the applicator. Each group’s own adhesive composite was applied to the base of the brackets and placed in the correct position on the tooth. Excess composite protruding from the bracket edges was removed with a probe. Light was applied to the brackets with a 3M EspeElipar S10 (3M ESPE Dental Products) light source at a light intensity of 1200 mW/cm^2^ and a wavelength of 430–480 nm, 10 s each from the mesial and distal directions, for a total of 20 s [24]. In the study, the brackets were subjected to shear bond tests after being stored in distilled water at room temperature for 24 h after bonding [25].

### 2.7. Performing Shear Bond Strength Tests

**Figure 2:**
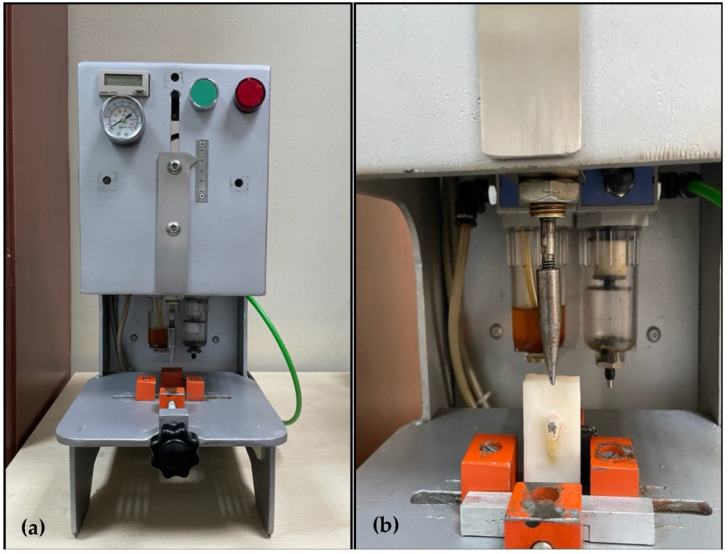
General representation of the fatigue machine (**a**) and the sample testing position (**b**).

**Figure 3:**
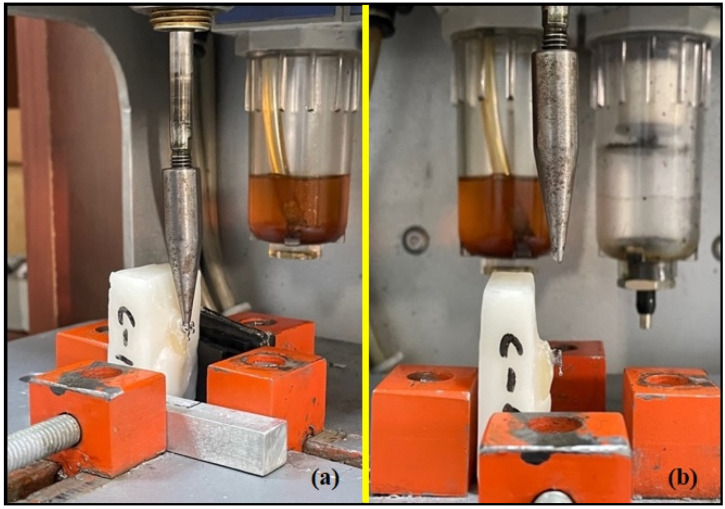
Checking the angle between the bracket groove and the metal nozzle tip from the lateral (**a**) and from the frontal (**b**).

In order to evaluate the shear bond strengths of the samples, cycling testing was conducted in a closed-loop controlled, homemade pneumatic-based fatigue machine with a capacity of 10 N and a crosshead speed of 300 mm/min. This velocity-controlled machine consisted of electrical actuators and a counter for the uniaxial cyclic loading and unloading tests of orthodontic brackets. While testing each sample, attention was paid to ensure that the angle between the metal nozzle tip and the bracket groove was 90°. By creating a fixing mechanism for each acrylic block, a 10 N stroke was applied and the number of strokes for each bracket failure was determined (see Figure 2 and Figure 3).

### 2.8. Statistical Analysis

Statistical analysis of the data in the study was performed using the IBM SPSS (version 28.0; SPSS, Chicago, IL, USA) program. Mean, standard deviation, median, lowest, highest, frequency, and ratio values were used in the descriptive statistics of the data. The distribution of variables was determined with the Kolmogorov–Smirnov test. As the data were not normally distributed, the Kruskal–Wallis test was performed to determine whether there was a statistical difference between the groups. The Mann–Whitney U test was used for the pair-wise comparison of the groups. The statistical significance level was determined to be *p* < 0.05.

## 3. Results

Statistical analysis was conducted using the Kolmogorov–Smirnov test. Additionally, statistically significant differences were found between the groups according to the Kruskal–Wallis and Mann–Whitney U tests (*p* < 0.05).

**Figure 4:**
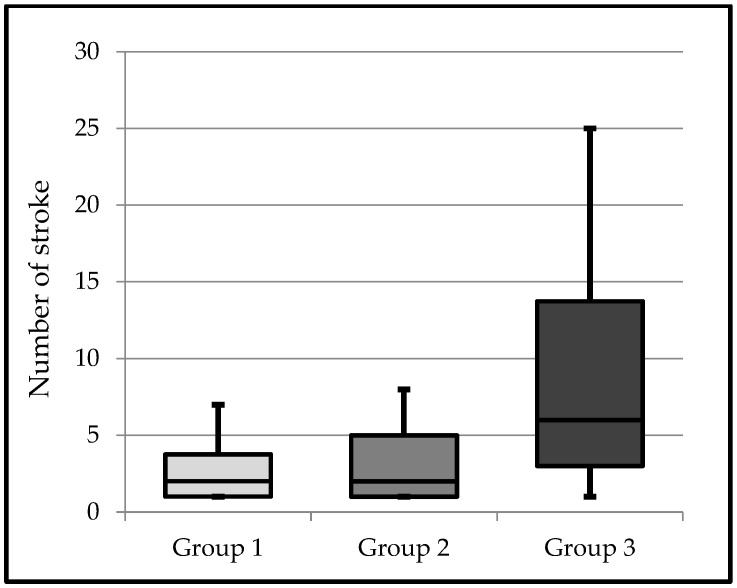
Box plot of the numbers of shear strokes.

In Group 1, the mean number of shear strokes was the lowest, at 2.8, and in Group 3, the average number of shear strokes was the highest, at 8.4. In Group 2, the average number of shear strokes was measured, at 3.1. The mean, standard deviation, median, lowest, and highest values of the bracket shear stroke numbers of the groups are shown in Table 2. In Figure 4, the bracket shear stroke numbers of the groups are shown in a box plot.

The numbers of shear strokes of the brackets in Group 3 were found to be statistically significantly higher than those in Group 1 and Group 2 (*p* < 0.05). There was no statistically significant difference between the numbers of shear stroke hits of brackets in Group 1 and Group 2 (*p* > 0.05).

Pairwise comparisons between the groups performed using the Mann–Whitney U test are shown in Table 3.

During the shear bond tests, a total of six samples, one in Group 1, one in Group 2, and four in Group 3, showed very high numbers of shear strokes. Therefore, these samples were renewed and resubjected to the shear bond tests.

## 4. Discussion

Various adhesive agents have been developed for the bonding of orthodontic brackets. The pioneering work has been instrumental in developing the procedures and materials that led to today’s standards in orthodontic adhesives. Acid etching, self-curing composite resins, glass ionomer cements, and visible light-curing adhesives were developed from these early efforts. Technologies that use new materials are constantly evolving to improve the quality of the bonds between brackets and teeth or artificial surfaces [26].

In the literature, there have been studies on the shear, tensile, and torsion strengths conducted in in vitro orthodontic-bond-strength tests and their clinical techniques, material types, and appliance designs [11,27,28]. Universal testing machines, such as Zwicki and Instron, have been used for this purpose [29,30]. There are also differences in the techniques used for debonding, there are various force-application methods, such as wire loops, blades, and metal tips [23,31,32,33]. The crosshead speed of the testing machine varies between orthodontic bond strength studies. When the orthodontic literature is reviewed, it can be seen that the crosshead speeds for bond strength tests generally vary from 0.5 to 5 mm/min [34,35,36,37]. However, Çiçek et al. [23] examined the shear stroke numbers of brackets using a 300 mm/min crosshead speed in their 2020 study. Klocke et al. [38] showed that the crosshead speed varying between 0.1 and 5 mm/min did not have a significant effect on orthodontic bond strength tests. In the study, shear tests of brackets were carried out with a closed-loop controlled, low-cycle, 10 N-capacity fatigue machine with a crosshead speed of 300 mm/min and a metal nozzle tip attached to it.

In many studies in which orthodontic bracket shear tests were performed, the direction of force application was parallel to the bonding interface [21,39,40]. Elsaka et al. [19] carried out shear bond tests of brackets in three different modes. When the shear forces were applied to the short side of the brackets, a higher SBS value was measured compared with the long side. In addition, it has been reported that brackets receiving tensile forces show the lowest SBS values. Klocke and Kahle-Nieke [41] investigated the effect of different angle changes between +15° and −45° in the direction of application of the debonding force on SBS. The lowest average bond strength was found in the −45° group, while the highest average bond strength was found in the +15° group. In our study, care was taken to ensure that the bracket shear forces were perpendicular to the bracket groove.

Rameez et al. [42] investigated the SBS values of three different color-changing adhesive agents: Transbond^TM^ Plus (3M Unitek, USA), Grengloo^TM^, and Blugloo^TM^ (Ormco, USA). Transbond^TM^ Plus was used with Transbond^TM^ XT primer, while Grengloo^TM^ and Blugloo^TM^ were bonded with Ortho Solo^TM^ primer. As a result of the study, they reported that, although Transbond^TM^ Plus showed acceptable bond strength, it showed a lower value compared with the Grengloo^TM^ and Blugloo^TM^ groups. Although there was no significant difference between Grengloo^TM^ and Blugloo^TM^, they stated that all adhesive agents showed clinically acceptable bond strengths.

Priya and Jain [43] compared the bond strengths of metal brackets bonded with Transbond^TM^ XT composite and Grengloo^TM^ composite. The acid-etching method and Transbond^TM^ XT primer were used in both groups. They reported that, although the bond strength was slightly higher in the Grengloo^TM^ group compared with the Transbond XT^TM^ group, they did not find a significant difference.

Knaup et al. [44] evaluated the shear bond strengths of different adhesives. Transbond^TM^ XT, BrackFix (VOCO^®^ GmbH, Cuxhaven, Germany), and Grengloo^TM^ adhesives were considered clinically adequate and did not show a statistically significant difference. Meron glass ionomer cement (VOCO^®^ GmbH, Cuxhaven, Germany) showed significantly lower shear bond strength than the other adhesives.

Stefanski et al. [45] examined the bond strength of six different adhesive agents in dry and saliva-contaminated environments. Enlight LC (Ormco, Glendora, CA, USA) adhesive and Ortho Solo^TM^ primer, Grengloo^TM^ adhesive and Ortho Solo^TM^ primer, Light Bond (Reliance Orthodontic Products, Itasca, IL, USA) adhesive and Light Bond primer, Charisma (Heraeus Kulzer, Wehrheim, Germany) adhesive and Gluma Self Etch (Heraeus Kulzer, Wehrheim, Germany) primer, SmartBond (Gestenco, Gothenburg, Sweden), and Transbond^TM^ XT adhesive and Transbond^TM^ MIP (3M) primer groups were compared. While no statistically significant difference was reported between the composite materials under dry conditions, it was reported that Smartbond showed a significantly lower bond strength.

Nimcharoensuk et al. [46] compared the SBS values of three different adhesive agents, namely Grengloo^TM^, Green Glue (Hangzhou Westlake Biomaterial, Hangzhou, China), and Transbond^TM^ XT. There was no significant difference in SBS between Grengloo^TM^ and Transbond^TM^ XT. They reported that Green Glue adhesive showed a statistically significantly lower SBS value compared with the others.

These different results seen in the past literature may be due to the numbers and types of sample teeth in the studies, the types of composite adhesives or primers used, the types of brackets used, the environments and storage times of the bonded samples, and the test application methods. In our study, the number of shear strokes recorded in Group 3 using Ortho Solo^TM^ primer and Grengloo^TM^ Adhesive composite was found to be significantly higher than those in Group 1 and Group 2.

Delavarian et al. [47] evaluated the SBS of the adhesive composites of Transbond^TM^ XT and Grengloo^TM^. Statistically significantly higher values were measured in the Grengloo^TM^ group compared with the Transbond^TM^ XT group. The authors reported that the high performance of the Grengloo^TM^ composite may have been due to the Ortho Solo^TM^ primer with which it was used. It has been reported that Ortho Solo^TM^ primer can increase the bond strength by showing impact-absorbing properties due to the glass fillers it contains.

Bayani et al. [48] measured the SBS values of different composite resins, including Resilience (Orthotechnology, Tampa, FL, USA), Grengloo^TM^, and Transbond^TM^ Plus. The highest values were recorded in the adhesive group of Grengloo^TM^. It has been reported that the chemical affinity of Grengloo^TM^ adhesive to some metal brackets may also contribute to the high SBS value of Grengloo^TM^ adhesive, as well as the glass filler content of the Ortho Solo^TM^ primer used in combination with it. Similarly, in our study, the superior shear strength performance of Grengloo^TM^ adhesive may have been related to its chemical affinity for metal brackets and the glass filler content of the Ortho Solo^TM^ primer.

Katırcıoğlu and Büyükbayraktar [49], in their study, compared Transbond^TM^ XT, Light Bond, BracePaste^®^, Nova Compo SF (Imicryl, Konya, Turkey), and Rely A Bond (Reliance, Itasca, USA) in terms of shear bond strength. It was reported that the Transbond^TM^ XT group showed a significantly higher value than the Nova Compo SF group. When Transbond^TM^ XT was compared with other groups, no significant difference was reported.

Shams et al. [50] evaluated three different adhesive agents in terms of SBS during two different time periods in their study. In the study, brackets were used with Transbond^TM^ XT, BracePaste^®^, and GoTo (Reliance Orthodontic Products, Itasca, IL, USA) adhesives with Transbond^TM^ XT primer. In tests performed after 24 h, there was no statistical difference between the GoTo and Transbond^TM^ XT groups, while the BracePaste^®^ group showed a significantly lower value. In the study, no statistical difference was found between Group 1 and Group 2 in terms of the numbers of shear strokes.

The main active ingredients of BracePaste^®^ are Bis-EMA, Ethoxylated bisphenol A-dimethacrylate, and TD: Tetramethylenedimethacrylate. The manufacturer claims that the bond strength of BracePaste^®^ is comparable to that of Transbond^TM^ XT, as the Bis-GMA and Quartz Silica components are similar. It has also been reported that the filler content of BracePaste^®^ is 70% and that of Transbond^TM^ XT is 80%. Therefore, the similar performances of BracePaste^®^ and Transbond^TM^ XT may depend on the ingredient properties [50].

There are several limitations to the results of this study that should be noted. First, it should be noted that the results are theoretical in nature and reflect an in vitro approach. Cyclic and unpredictable temperature changes in the oral cavity, which can change the material properties, cannot be fully transferred to in vitro approaches. However, in our study, care was taken to ensure that the bracket shear forces were perpendicular to the bracket groove. Second, in order to determine the actual shear bond strength, only pure shear force must be applied directly to the bracket–adhesive interface [51]. This is a difficult practice to achieve, and there is inevitably some distance from this point where force must be applied. On the other hand, factors such as patients’ dietary habits and the polymerization time mode may affect the mechanical properties of composite materials [52,53]. Despite all of the limitations, this study provides a basis for further research to provide clinical implications for bonding procedures by testing the shear bond strengths of different types of adhesive agents under cyclic loading conditions.

## 5. Conclusions

In this study, the statements below are concluded:

i. Different types of adhesive agents affect the shear bond strengths of brackets;
ii. Grengloo™ adhesive can be preferable in situations where high bond strength is required, as it shows a significantly higher number of shear strokes than the Transbond™ XT Light Cure Adhesive Paste and BracePaste^®^ adhesives;
iii. BracePaste^®^ adhesive and Transbond™ XT Light Cure Adhesive Paste can be used clinically as alternatives to each other because they exhibit similar numbers of shear strokes;
iv. Considering the limitations of the study, it may seem that there is a need for further in vivo studies.

## Figures and Tables

**Table 1:** Compositions and weight percentages of adhesive agents used in the study.

Groups	Manufacturer	Adhesive Agents	Composition of Adhesive Agent	wt%
Group 1	3M Unitek, Monrovia, CA, USA	Transbond™ XT Primer	Bisphenol A Diglycidly Ether Dimethacrylate (BİSGMA)	45–55
			Triethylene Glycol Dimethacrylate (TEGDMA)	45–55
			4-(Dimethylamino)-Benzeneethanol	<0.5
	3M Unitek, Monrovia, CA, USA	Transbond™ XT Light Cure Adhesive	Silane Treated Quartz	70–80
			Bisphenol A Diglycidyl Ether Dimethacrylate (BİSGMA)	10–20
			Bisphenol A Dimethacrylate	5–10
			Silane Treated Silica	<2
			Diphenyliodonium Hexafluorophosphate	<1
			Triphenylantimony	<1
Group 2	American Orthodontics, Sheboygan, WI, USA	BracePaste^®^ MTP Primer	Ethanol	20–50
			2-Hydroxyethyl methacrylate	10–25
			2-Hydroxy-1,3-propanediyl bismethacrylate	2.5–10
	American Orthodontics, Sheboygan, WI, USA	BracePaste^®^ Adhesive	Ethoxylated Bisphenol A Dimethacrylate	2.5–12
			Tetramethylene Dimethacrylate	<3
			Diphenyl(2,4,6-trimethylbenzoyl)phosphine oxide	<3
Group 3	Ormco, Orange, CA, USA	Ortho Solo™ Primer	2-Hydroxyethyl methacrylate	30–60
			2-Hydroxy-1,3-propanediyl bismethacrylate	5–10
			Ethanol	1–5
			3-Trimethoxysilylpropyl methacrylate	1–5
			Disodium hexafluorosilicate	0.1–1
	Ormco, Glendora, CA, USA	Grengloo™ Adhesive	Bisphenol A ethoxylate dimethacrylate	≤2.9
			Silica, amorphous, fumed, and cryst.-free	≤1.9
			2,3-Epoxypropyl methacrylate	<1
			Propylidynetrimethanol, ethoxylated, esters with acrylic acid	<1
			7,7,9 (or 7,9,9)-Trimethyl-4,13-dioxo-3,14-dioxa-5,12-diazahexadecane-1,16-diyl bismethacrylate	<1
			Phenyl bis (2,4,6-trimethylbenzoyl)-phosphine oxide	<1
			Bisphenol A	≤0.3

According to the manufacturer, the compositions of the adhesive agents used were provided by the 3M Unitek’s Safety Data Sheet for Group 1, American Orthodontics’ Safety Data Sheet for Group 2, and Ormco’s Safety Data Sheet for Group 3.

**Table 2:** The numbers of shear strokes applied to the brackets.

Test Group	Sample	Mean Number of Strokes	Median	Standard Deviation	Min	Max	*p*
Group 1	20	2.8	2	1.8	1.0	7.0	
Group 2	20	3.1	2	2.3	1.0	8.0	0.002 *
Group 3	20	8.4	6	6.9	1.0	25.0	

Min: minimum, Max: maximum, *p*: significance level, *: *p* < 0.05.

**Table 3:** Pairwise comparison results of the groups in the study.

Groups	N	Mean Rank	Sum of Ranks	*p* ^a^
Group 1	20	20.48	409.50	0.989
Group 2	20	20.53	410.50	
Total	40			
Group 1	20	14.78	295.50	0.002 *
Group 3	20	26.23	524.50	
Total	40			
Group 2	20	15.03	300.50	0.003 *
Group 3	20	25.98	519.50	
Total	40			

N: sample size, ^a^: Mann–Whitney U test, *p*: significance level, * *p* < 0.05.

## Data Availability

All data supporting the results of this study are included within the article.

## References

[1] Çiçek O., Özkalaycı N. (2018). Orthodontic Brackets; Part II. J. Int. Dent. Sci..

[2] Gange P. (2015). The Evolution of Bonding in Orthodontics. Am. J. Orthod. Dentofac. Orthop..

[3] Buonocore M.G. (1955). A Simple Method of Increasing the Adhesion of Acrylic Filling Materials to Enamel Surfaces. J. Dent. Res..

[4] Newman G.V. (1965). Epoxy Adhesives for Orthodontic Attachments: Progress Report. Am. J. Orthod..

[5] Bakhadher W., Halawany H., Talic N., Abraham N., Jacob V. (2015). Factors Affecting the Shear Bond Strength of Orthodontic Brackets—A Review of In Vitro Studies. Acta Med. (Hradec Kral.).

[6] Alzainal A.H., Majud A.S., Al-Ani A.M., Mageet A.O. (2020). Orthodontic Bonding: Review of the Literature. Int. J. Dent..

[7] Khan H., Mheissen S., Iqbal A., Jafri A.R., Alam M.K. (2022). Bracket Failure in Orthodontic Patients: The Incidence and the Influence of Different Factors. BioMed Res. Int..

[8] Prasad M., Mohamed S., Nayak K., Shetty S.K., Talapaneni A.K. (2014). Effect of Moisture, Saliva, and Blood Contamination on the Shear Bond Strength of Brackets Bonded with a Conventional Bonding System and Self-Etched Bonding System. J. Nat. Sci. Biol. Med..

[9] Türköz Ç., Ulusoy Ç. (2012). Evaluation of Different Enamel Conditioning Techniques for Orthodontic Bonding. Korean J. Orthod..

[10] Pickett K.L., Sadowsky P.L., Jacobson A., Lacefield W. (2001). Orthodontic in Vivo Bond Strength: Comparison with in Vitro Results. Angle Orthod..

[11] Trakinienė G., Petravičiūtė G., Smailienė D., Narbutaitė J., Armalaitė J., Lopatienė K., Šidlauskas A., Trakinis T. (2019). Impact of Fluorosis on the Tensile Bond Strength of Metal Brackets and the Prevalence of Enamel Microcracks. Sci. Rep..

[12] Reynolds I.R. (1975). A Review of Direct Orthodontic Bonding. Br. J. Orthod..

[13] Lyons L.K., English J.D., Ontiveros J.C., Bussa Jr H.I., Harris L.M., Laman S., Kasper F.K. (2019). In Vitro Shear Testing of Orthodontic Bonding to Lithium Disilicate Ceramic. J. Cosmet. Dent..

[14] Su M., Lai E.H.-H., Chang J.Z.-C., Chen H.-J., Chang F.H.-F., Chiang Y.-C., Lin C.-P. (2012). Effect of Simulated Debracketing on Enamel Damage. J. Formos. Med. Assoc..

[15] Brown K. (2009). The Impact of Bonding Material on Bracket Failure. Vital.

[16] Almosa N., Zafar H. (2018). Incidence of Orthodontic Brackets Detachment during Orthodontic Treatment: A Systematic Review. Pak. J. Med. Sci..

[17] Menini A., Cozzani M., Sfondrini M.F., Scribante A., Cozzani P., Gandini P. (2014). A 15-Month Evaluation of Bond Failures of Orthodontic Brackets Bonded with Direct versus Indirect Bonding Technique: A Clinical Trial. Prog. Orthod..

[18] Sukhia R.H., Sukhia H.R., Azam S.I., Nuruddin R., Rizwan A., Jalal S. (2019). Predicting the Bracket Bond Failure Rate in Orthodontic Patients: A Retrospective Cohort Study. Int. Orthod..

[19] Elsaka S.E., Hammad S.M., Ibrahim N.F. (2014). Evaluation of Stresses Developed in Different Bracket-Cement-Enamel Systems Using Finite Element Analysis with in Vitro Bond Strength Tests. Prog. Orthod..

[20] Eliades T., Brantley W.A. (2000). The Inappropriateness of Conventional Orthodontic Bond Strength Assessment Protocols. Eur. J. Orthod..

[21] Sha H.N., Choi S.H., Yu H.S., Hwang C.J., Cha J.Y., Kim K.M. (2018). Debonding Force and Shear Bond Strength of an Array of CAD/CAM-Based Customized Orthodontic Brackets, Placed by Indirect Bonding- an in Vitro Study. PLoS ONE.

[22] Farret M.M., Gonçalves T.S., de Lima E.M.S., de Menezes L.M., Oshima H.M.S., Kochenborger R., Mota Freitas M.P. (2010). The Influence of the Methodological Variables on the Shear Bond Strength. Dent. Press J. Orthod..

[23] Cicek O., Ozkalayci N., Yetmez M. (2020). Mean Shearing Stroke Frequency of Orthodontic Brackets under Cycling Loading: An In Vitro Study. Materials.

[24] Oz A.A., Oz A.Z., Arici S. (2016). In-Vitro Bond Strengths and Clinical Failure Rates of Metal Brackets Bonded with Different Light-Emitting Diode Units and Curing Times. Am. J. Orthod. Dentofac. Orthop..

[25] Ansari M.Y., Agarwal D.K., Gupta A., Bhattacharya P., Ansar J., Bhandari R. (2016). Shear Bond Strength of Ceramic Brackets with Different Base Designs: Comparative in-Vitro Study. J. Clin. Diagn. Res..

[26] Sharma S., Tandon P., Nagar A., Singh G.P., Singh A., Chugh V.K. (2014). A Comparison of Shear Bond Strength of Orthodontic Brackets Bonded with Four Different Orthodontic Adhesives. J. Orthod. Sci..

[27] González-Serrano C., Baena E., Fuentes M.V., Albaladejo A., Míguez-Contreras M., Lagravère M.O., Ceballos L. (2019). Shear Bond Strength of a Flash-Free Orthodontic Adhesive System after Thermal Aging Procedure. J. Clin. Exp. Dent..

[28] Franz A., Raabe M., Lilaj B., Dauti R., Moritz A., Müßig D., Cvikl B. (2019). Effect of Two Different Primers on the Shear Bond Strength of Metallic Brackets to Zirconia Ceramic. BMC Oral Health.

[29] Schauseil M., Blöcher S., Hellak A., Roggendorf M.J., Stein S., Korbmacher-Steiner H. (2016). Shear Bond Strength and Debonding Characteristics of a New Premixed Self-Etching with a Reference Total-Etch Adhesive. Head Face Med..

[30] Mehta A.S., Evans C.A., Viana G., Bedran-Russo A., Galang-Boquiren M.T.S. (2016). Bonding of Metal Orthodontic Attachments to Sandblasted Porcelain and Zirconia Surfaces. BioMed Res. Int..

[31] Pham D., Bollu P., Chaudhry K., Subramani K. (2017). Comparative Evaluation of Orthodontic Bracket Base Shapes on Shear Bond Strength and Adhesive Remnant Index: An in Vitro Study. J. Clin. Exp. Dent..

[32] Kwak J.-Y., Jung H.-K., Choi I.-K., Kwon T.-Y. (2016). Orthodontic Bracket Bonding to Glazed Full-Contour Zirconia. Restor. Dent. Endod..

[33] Al-Nafori M.K., Elshal M.G., Refai W.M. (2017). The Effect of Incorporating Gold and Silver Nanoparticles in Orthodontic Adhesive System on Bond Strength of Orthodontic Bracket. EC Dent. Sci..

[34] Khanal P.P., Shrestha B.K., Yadav R., Gupta S.P. (2021). A Comparative Study on the Effect of Different Methods of Recycling Orthodontic Brackets on Shear Bond Strength. Int. J. Dent..

[35] Dias F.M.C.S., Pinzan-Vercelino C.R.M., de Tavares R.R.J., de Gurgel J.A., Bramante F.S., Fialho M.N.P. (2015). Evaluation of an Alternative Technique to Optimize Direct Bonding of Orthodontic Brackets to Temporary Crowns. Dent. Press J. Orthod..

[36] Goymen M., Topcuoglu T., Topcuoglu S., Akin H. (2015). Effect of Different Temporary Crown Materials and Surface Roughening Methods on the Shear Bond Strengths of Orthodontic Brackets. Photomed. Laser Surg..

[37] Pinho M.M., Manso M.C., Martin C., Souza J.C.M., Almeida R.F., Ferreira A.P. (2017). Adhesion Strength of Orthodontic Brackets to Acrylic Surfaces. A Systematic Review on in Vitro Studies. Rev. Port. Estomatol. Med. Dent. Cir. Maxilofac..

[38] Klocke A., Kahl-Nieke B. (2005). Influence of Cross-Head Speed in Orthodontic Bond Strength Testing. Dent. Mater..

[39] Mohammadi A., Pourkhameneh S., Sadrhaghighi A.H. (2018). The Effect of Different Force Magnitudes for Placement of Orthodontic Brackets on Shear Bond Strength, in Three Adhesive Systems. J. Clin. Exp. Dent..

[40] Shaik J.A., Reddy R.K., Bhagyalakshmi K., Shah M.J., Madhavi O., Ramesh S.V. (2018). In Vitro Evaluation of Shear Bond Strength of Orthodontic Brackets Bonded with Different Adhesives. Contemp. Clin. Dent..

[41] Klocke A., Kahl-Nieke B. (2006). Effect of Debonding Force Direction on Orthodontic Shear Bond Strength. Am. J. Orthod. Dentofac. Orthop..

[42] Rameez M., Kiran H., Alle R.S., Bharathi V.S., Dharmesh H.S. (2020). Comparison of SBS of Colour Changing Adhesives-Transbond Plus, Blugloo, Grengloo. J. Adv. Med. Dent. Sci. Res..

[43] Priya B., Jain R.K. (2018). Comparison of Shear Bond Strength of Different Light Cure Orthodontic Adhesives—An In Vitro Study. Res. J. Pharm. Technol..

[44] Knaup I., Böddeker A., Tempel K., Weber E., Bartz J.R., Rückbeil M.V., Craveiro R.B., Wagner Y., Wolf M. (2020). Analysing the Potential of Hydrophilic Adhesive Systems to Optimise Orthodontic Bracket Rebonding. Head Face Med..

[45] Stefański T., Kloc-Ptaszna A., Postek-Stefańska L. (2019). Bond Strength of Orthodontic Adhesives to Dry and Saliva-Moistened Enamel—A Comparative in Vitro Study. Arch. Mater. Sci. Eng..

[46] Nimcharoensuk K., Anuwongnukroh N., Dechkunakorn S. (2018). Shear Bond Strength of Three Light-Cured Orthodontic Adhesives. Key Eng. Mater..

[47] Delavarian M., Rahimi F., Mohammadi R., Imani M. (2019). Shear Bond Strength of Ceramic and Metal Brackets Bonded to Enamel Using Color-Change Adhesive. Dent. Res. J..

[48] Bayani S., Ghassemi A., Manafi S., Delavarian M. (2015). Shear Bond Strength of Orthodontic Color-Change Adhesives with Different Light-Curing Times. Dent. Res. J..

[49] Katırcıoğlu A., Büyükbayraktar Z. (2022). Evaluation of the Shear Bond Strength of Light-Cured and Self-Cured Orthodontic Adhesives. Orthod. Forum.

[50] Shams S., Abela S., Andiappan M., Hajiheshmati A., Bister D. (2020). Shear Bond Strengths of 3 Commonly Used Orthodontic Adhesives. Dentistry.

[51] Fox N.A., McCabe J.F., Buckley J.G. (1994). A Critique of Bond Strength Testing in Orthodontics. Br. J. Orthod..

[52] Szalewski L., Wójcik D., Sofińska-Chmiel W., Kuśmierz M., Różyło-Kalinowska I. (2023). How the Duration and Mode of Photopolymerization Affect the Mechanical Properties of a Dental Composite Resin. Materials.

[53] Szalewski L., Wójcik D., Bogucki M., Szkutnik J., Różyło-Kalinowska I. (2021). The Influence of Popular Beverages on Mechanical Properties of Composite Resins. Materials.

